# Ten Simple Rules for Writing a Postdoctoral Fellowship

**DOI:** 10.1371/journal.pcbi.1004934

**Published:** 2016-07-14

**Authors:** Ke Yuan, Lei Cai, Siu Ping Ngok, Li Ma, Crystal M. Botham

**Affiliations:** 1 Division of Pulmonary and Critical Care Medicine, Department of Medicine, Stanford University, Stanford, California, United States of America; 2 Department of Materials Science and Engineering, Stanford Neuroscience Institute, Stanford University, Stanford, California, United States of America; 3 Division of Hematology, Oncology, Stem Cell Transplantation, and Cancer Biology, Department of Pediatrics, Stanford University School of Medicine, Stanford, California, United States of America; 4 Asian Liver Center and Department of Surgery, Stanford University, Stanford, California, United States of America; 5 Stanford Biosciences Grant Writing Academy, Stanford University, Stanford, California, United States of America; Whitehead Institute, UNITED STATES

## Introduction

Postdoctoral fellowships support research, and frequently career development training, to enhance your potential to becoming a productive, independent investigator. Securing a fellowship sends a strong signal that you are capable of conducting fundable research and will likely lead to successes with larger grants. Writing a fellowship will also increase your productivity and impact because you will learn and refine skills necessary to articulate your research priorities. However, competition is fierce and your fellowship application needs to stand out among your peers as realistic, coherent, and compelling. Also, reviewers, a committee of experts and sometimes non-experts, will scrutinize your application, so anything less than polished may be quickly eliminated. We have drawn below ten tips from our experiences in securing postdoctoral fellowships to help as you successfully tackle your proposal.

## Rule 1: Start Early and Gather Critical Information

Crafting a competitive fellowship can take 6–9 months, so it is imperative that you start early. You may even want to start looking for postdoctoral fellowships before you finish your doctoral degree. Compile a comprehensive list of fellowships that you can apply to. This list should include key information to organize your game plan for applying, including Sponsor (agency sponsoring the fellowship) name; URL for funding information; Sponsor deadlines; and any other requirements or critical information.

To find suitable fellowships, start by asking your faculty mentor(s), laboratory colleagues, and recent alumni about their experiences applying for fellowships. Federal agencies in the United States, such as the National Institute of Health (NIH) and National Science Foundation (NSF); foreign governmental agencies; and other organizations, such as societies, foundations, and associations, often solicit fellowship applications. Additionally, many institutions offer internally supported fellowships as well as institutional research training grants.

Once you have an exhaustive list of fellowships you are eligible for, start gathering critical information that you can use to inform your writing. Read the fellowship instructions completely and identify the review criteria. Investigate the review process; NIH’s Center for Scientific Review reviews grant applications for scientific merit and has a worthwhile video about the Peer Review Process [1]. Sometimes Sponsors offer notification alerts about upcoming funding opportunities, deadlines, and updated policies, so make sure to sign up for those when offered. Also, gather previously submitted applications and reviewers’ comments for the fellowships you will to apply to. Both funded and unfunded applications are useful. Sometimes Sponsors make available funded abstracts like NIH’s Research Portfolio Online Reporting Tools (RePORT), and these provide critical information about the scope of funded projects.

Many institutions have internal policies and processes that are required before a proposal can be submitted to a Sponsor. These requirements can include waivers to assess eligibility and internal deadlines (five business day internal deadlines are standard), so make sure you also gather relevant information about any internal policies and processes required by your institution.

## Rule 2: Create a Game Plan and Write Regularly

Writing a compelling fellowship takes time, a lot of time, which is challenging to balance with a hectic laboratory schedule, other responsibilities, and family obligations. To reduce stress, divide the fellowship requirements into smaller tasks by creating a detailed timeline with goals or milestones. Having a game plan with daily and/or weekly goals will also help you avoid procrastination. Make sure you are writing regularly (i.e., daily or every other day) to establish an effective writing practice. This will increase your productivity and reduce your anxiety because writing will become a habit. It is also important to make your writing time non-negotiable so other obligations or distractions don’t impede your progress.

## Rule 3: Find Your Research Niche

It is crucial that you have a deep awareness of your field so you can identify critical knowledge gaps that will significantly move your field forward when filled. Keep a list of questions or problems inherent to your field and update this list after reading germane peer-reviewed and review articles or attending seminars and conferences. Narrow down and focus your list through discussions with your mentor(s), key researchers in your field, and colleagues. Because compelling projects often combine two seemingly unrelated threads of work to challenge and shift the current research or clinical practice paradigms, it is important to have a broad familiarity with the wider scientific community as well. Seek opportunities to attend seminars on diverse topics, speak with experts, and read broadly the scientific literature. Relentlessly contemplate how concepts and approaches in the wider scientific community could be extended to address critical knowledge gaps in your field. Furthermore, develop a few of your research questions by crafting hypotheses supported by the literature and/or preliminary data. Again, share your ideas with others, i.e., mentor(s), other scientists, and colleagues, to gauge interest in the significance and innovation of the proposed ideas. Remember, because your focus is on writing a compelling fellowship, make sure your research questions are also relevant and appropriate for the missions of the sponsoring agencies.

## Rule 4: Use Your Specific Aims Document as Your Roadmap

A perfectly crafted Specific Aims document, usually a one-page description of your plan during the project period, is crucial for a compelling fellowship because your reviewers will read it! In fact, it is very likely your Specific Aims will be the first document your reviewers will read, so it is vital to fully engage the reviewers’ interest and desire to keep reading. The Specific Aims document must concisely answer the following questions:

- *Is the research question important?* Compelling proposals often tackle a particular gap in the knowledge base that, when addressed, significantly advance the field.
- *What is the overall goal?* The overall goal defines the purpose of the proposal and must be attainable regardless of how the hypothesis tests.
- *What specifically will be done?* Attract the reviewers’ interest using attention-getting headlines. Describe your working hypothesis and your approach to objectively test the hypothesis.
- *What are the expected outcomes and impact?* Describe what the reviewers can expect after the proposal is completed in terms of advancement to the field.

A draft of your Specific Aims document is ideal for eliciting feedback from your mentor(s) and colleagues because evaluating a one-page document is not an enormous time investment on part of the person giving you feedback. Plus, you don’t want to invest time writing a full proposal without knowing the proposal’s conceptual framework is compelling. When you are ready to write the research plan, your Specific Aims document then provides a useful roadmap.

As you are writing (and rewriting) your Specific Aims document, it is essential to integrate the Sponsor’s goals for that fellowship funding opportunity. Often goals for a fellowship application include increasing the awardee’s potential for becoming an independent investigator, in which case an appropriate expected outcome might be that you mature into an independent investigator.

We recommend reading The Grant Application Writer’s Workbook (www.grantcentral.com) [2] because it has two helpful chapters on how to write a persuasive Specific Aims document, as well as other instructive chapters. Although a little formulaic, the Workbook’s approach ensures the conceptual framework of your Specific Aims document is solid. We also advise reading a diverse repertoire of Specific Aims documents to unearth your own style for this document.

## Rule 5: Build a First-Rate Team of Mentors

Fellowship applications often support mentored training experiences; therefore, a strong mentoring team is essential. Remember, reviewers often evaluate the qualifications and appropriateness of your mentoring team. The leader of your mentoring team should have a track record of mentoring individuals at similar stages as your own as well as research qualifications appropriate for your interests. Reviewers will also often consider if your mentor can adequately support the proposed research and training because fellowship applications don’t always provide sufficient funds. It is also useful to propose a co-mentor who complements your mentor’s qualifications and experiences. You should also seek out other mentors at your institution and elsewhere to guide and support your training. These mentors could form an advisory committee, which is required for some funding opportunities, to assist in your training and monitor your progress. In summary, a first-rate mentoring team will reflect the various features of your fellowship, including mentors who augment your research training by enhancing your technical skills as well as mentors who support your professional development and career planning.

As you develop your fellowship proposal, meet regularly with your mentors to elicit feedback on your ideas and drafts. Your mentors should provide feedback on several iterations of your Specific Aims document and contribute to strengthening it. Recruit mentors to your team who will also invest in reading and providing feedback on your entire fellowship as an internal review before the fellowship’s due date.

You also want to maintain and cultivate relationships with prior mentors, advisors, or colleagues because fellowships often require three to five letters of reference. A weak or poorly written letter will negatively affect your proposal’s fundability, so make sure your referees will write a strong letter of recommendation and highlight your specific capabilities.

## Rule 6: Develop a Complete Career Development Training Plan

Most fellowships support applicants engaged in training to enhance their development into a productive independent researcher. Training often includes both mentored activities, e.g., regular meetings with your mentor(s), as well as professional activities, e.g., courses and seminars. It is important that you describe a complete training plan and justify the need for each training activity based on your background and career goals.

When developing this plan, it is helpful to think deeply about your training needs. What skills or experiences are missing from your background but needed for your next career stage? Try to identify three to five training goals for your fellowship and organize your plan with these goals in mind. Below are sample activities:

- Regular (weekly) one-on-one meetings with mentor(s)
- Biannual meeting with advisory committee
- Externship (few weeks to a few months) in a collaborator’s laboratory to learn a specific technique or approach
- Courses (include course # and timeline) to study specific topics or methods
- Seminars focused on specific research areas
- Conferences to disseminate your research and initiate collaborations
- Teaching or mentoring
- Grant writing, scientific writing, and oral presentation courses or seminars
- Opportunities for gaining leadership roles
- Laboratory management seminars or experiences

## Rule 7: STOP! Get Feedback

Feedback is critical to developing a first-class proposal. You need a wide audience providing feedback because your reviewers will likely come from diverse backgrounds as well. Be proactive in asking for feedback from your mentor, colleagues, and peers. Even non-scientists can provide critical advice about the clarity of your writing. When eliciting feedback, inform your reviewer of your specific needs, i.e., you desire broader feedback on overall concepts and feasibility or want advice on grammar and spelling. You may also consider hiring a professional editing and proofreading service to polish your writing.

Some fellowships have program staff, such as the NIH Program Officers, who can advise prospective applicants. These individuals can provide essential information and feedback about the programmatic relevance of your proposal to the Sponsor’s goals for that specific fellowship application. Approaching a Program Officer can be daunting, but reading the article “What to Say—and Not Say—to Program Officers” can help ease your anxiety [3].

## Rule 8: Tell a Consistent and Cohesive Story

Fellowship applications are often composed of numerous documents or sections. Therefore, it is important that all your documents tell a consistent and cohesive story. For example, you might state your long term goal in the Specific Aims document and personal statement of your biosketch, then elaborate on your long term goal in a career goals document, so each of these documents must tell a consistent story. Similarly, your research must be described consistently in your abstract, Specific Aims, and research strategy documents. It is important to allow at least one to two weeks of time after composing the entire application to review and scrutinize the story you tell to ensure it is consistent and cohesive.

## Rule 9: Follow Specific Requirements and Proofread for Errors and Readability

Each fellowship application has specific formats and page requirements that must be strictly followed. Keep these instructions and the review criteria close at hand when writing and revising. Applications that do not conform to required formatting and other requirements might be administratively rejected before the review process, so meticulously follow all requirements and guidelines.

Proofread your almost final documents for errors and readability. Errors can be confusing to reviewers. Also, if the documents have many misspellings or grammar errors, your reviewers will question your ability to complete the proposed experiments with precision and accuracy. Remove or reduce any field-specific jargon or acronyms. Review the layout of your pages and make sure each figure or table is readable and well placed. Use instructive headings and figure titles that inform the reviewers of the significance of the next paragraph(s) or results. Use bolding or italics to stress key statements or ideas. Your final documents must be easy to read, but also pleasing, so your reviewers remain engaged.

## Rule 10: Recycle and Resubmit

Fellowships applications frequently have similar requirements, so it is fairly easy to recycle your application or submit it to several different funding opportunities. This can significantly increase your odds for success, especially if you are able to improve your application with each submission by tackling reviewers’ comments from a prior submission. However, some Sponsors limit concurrent applications to different funding opportunities, so read the instructions carefully.

Fellowship funding rates vary but, sadly, excellent fellowships may go unfunded. Although this rejection stings, resubmitted applications generally have a better success rate than original applications, so it is often worth resubmitting. However, resubmitting an application requires careful consideration of the reviewers’ comments and suggestions. If available, speak to your Program Officers because he or she may have listened to the reviewers’ discussion and can provide a unique prospective or crucial information not included in the reviewers’ written comments. Resubmitted fellowships are many times allowed an additional one- to two-page document to describe how you addressed the reviewers’ comments in the revised application, and this document needs to be clear and persuasive.

## Conclusion

The ten tips we provide here will improve your chances of securing a fellowship and can be applied to other funding opportunity announcements like career development awards (i.e., NIH K Awards). Regardless of funding outcomes, writing a fellowship is an important career development activity because you will learn and refine skills that will enhance your training.
